# WHO’s 2026 emergency appeal and global health security

**DOI:** 10.1186/s12992-026-01211-1

**Published:** 2026-04-17

**Authors:** Yusuf Hared Abdi, Sharmake Gaiye Bashir, Walid Abdulkadir Osman, Ahmed Abdinasir Abdulle

**Affiliations:** 1 Faculty of medicine and Health science, Hormuud University, Mogadishu, Somalia; 2 Center for Health Research and Innovation, Somali National University, Mogadishu, Somalia; 3 Faculty of Health Science, Salaam University, Mogadishu, Somalia; 4 Faculty of Health Science, Mogadishu University, Mogadishu, Somalia; 5 Mogadishu Institute of Health, Mogadishu, Somalia

**Keywords:** WHO emergency appeal, Humanitarian financing, Global health security

## Abstract

The World Health Organization’s 2026 global appeal seeks nearly US$1 billion to sustain life-saving health interventions amid escalating humanitarian crises. This Letter highlights persistent funding shortfalls affecting fragile and conflict-affected states, including Somalia, Sudan, and Yemen, where service suspensions and outbreaks have compounded morbidity and mortality. Despite WHO and partners reaching millions in 2025, financing remains insufficient, jeopardizing maternal and child health, outbreak response, and health system resilience. We argue that predictable, front-loaded financing is critical to support local actors, strengthen health systems, and safeguard global health security. Integrating climate-resilient infrastructure, One Health surveillance, and equity-focused strategies can mitigate preventable deaths and stabilize vulnerable populations. The 2026 appeal represents a strategic investment in global health and security, and by mobilizing front-loaded, flexible support alongside sustained assessed contributions requires urgent international solidarity and collective action.

The World Health Organization’s (WHO) launch of its 2026 global appeal on February 3, 2026, seeking nearly US$1 billion, underscores the urgent imperative to sustain life-saving health interventions amid escalating humanitarian crises [1]. This appeal targets 36 emergencies, including 14 Grade 3 crises— the highest organizational response level—such as protracted conflicts in Afghanistan, the Democratic Republic of the Congo, Somalia, Sudan, Yemen, and Ukraine, alongside outbreaks of cholera and mpox. In 2025, WHO and partners reached 30 million people, delivering 53 million health consultations, vaccinating 5.3 million children, and supporting over 8,000 facilities and 1,370 mobile clinics, yet funding shortfalls limited coverage to one-third of the 81 million targeted [1].

Global humanitarian financing has contracted sharply, falling below 2016 levels in 2025, exacerbating health system collapses in crisis settings [2]. In Somalia, a protracted conflict and drought-affected Grade 3 emergency, funding cuts led to the suspension of services in eight hospitals, funding cuts led to the suspension of services in eight hospitals, 40 primary facilities, and 16 mobile teams across 21 districts, denying care to 350,000 people and contributing to diphtheria outbreaks (1,811 cases, 89 deaths by August 2025) [3–5]. Maternal mortality—one of the world’s highest—persists amid conflict, drought, and displacement affecting 5.9 million people, with the 2025 Humanitarian Needs and Response Plan only 20% funded [6]. Similarly, Sudan’s conflict has triggered regional health crises with mass displacement, collapsing infrastructure, and heightened outbreak risks [7], while Yemen’s prolonged war leaves 19.6 million without basic services, forcing facility closures [7].

While WHO’s assessed contributions from Member States offer a critical baseline of predictable financing, they currently represent less than one-fifth of WHO’s overall budget and are often insufficient or delayed [1], constraining the Organization’s ability to rapidly scale operations in acute crises. In contrast, the 2026 health emergency appeal is designed to mobilize front-loaded resources that can be disbursed within weeks rather than months, enabling WHO and partners to pre-position supplies, preserve surge capacity and sustain life-saving services in high-risk settings such as Somalia, Sudan and Yemen. Early and flexible appeal funding can bridge the gap between slow or partial fulfilment of statutory contributions and the urgent, time-bound nature of humanitarian health needs, reducing the risk of service suspensions, preventable outbreaks and costly late-stage responses [8–10].

Calls for Member States to honour and increase their assessed contributions remain essential to correcting WHO’s structural underfunding and dependence on tightly earmarked voluntary funds. However, assessed contributions alone cannot provide the rapid, context-specific surge financing needed to manage Grade 3 emergencies and compounding shocks from conflict and climate events. Supporting the 2026 emergency appeal therefore complements efforts to strengthen the core budget: it channels additional, ideally unearmarked or softly earmarked resources, meaning flexible voluntary funds that are restricted only to broad priorities rather than individual projects, into the most under-served and crisis-affected settings, where delays in funding translate directly into excess mortality and heightened regional health security risks [10–12].

These converging crises—driven by climate change, protracted conflicts, and infectious threats—are overwhelming fragile health systems while donor fatigue and geopolitical shifts erode support. In this context, WHO’s coordination of more than 1,500 partners across 24 emergency settings remains indispensable for delivering trauma care, outbreak response, immunization, and reproductive health services. As Dr Tedros Adhanom Ghebreyesus has emphasized, fully financing the 2026 health emergency appeal is a strategic investment in global health security that helps restore dignity and stabilize communities on the path to recovery [13].

Prior appeals demonstrate impact: early funding averts escalation, containing outbreaks and reducing long-term costs, as evidenced in evaluations of WHO’s Grade 3 responses like Cyclone Idai [14]. Yet, persistent gaps—over 5,600 facilities curtailing services, affecting 53 million—signal dire underfunding at 40% below 2024 levels [13]. Renewed commitments, including unearmarked funds like Ireland’s Contingency Fund contributions, are vital for flexibility in the most severely affected, high-risk settings [13].

Policymakers and donors must prioritize front-loaded, predictable financing in tandem with meeting and increasing assessed contributions, to bridge the persistent gap between humanitarian health needs and available financing, and to empower local actors and national authorities at the response core [15]. In Somalia and other fragile, conflict-affected, and climate-vulnerable settings, integrating climate-resilient infrastructure, One Health surveillance, and equity-focused strategies can fortify health systems. Absent such solidarity, preventable deaths will mount, undermining global health security. The 2026 appeal demands collective action to affirm that no crisis is forsaken.

## Data Availability

Not applicable. All information used is derived from publicly available sources cited in the manuscript.
